# Breath-hold and free-breathing quantitative assessment of biventricular volume and function using compressed SENSE: a clinical validation in children and young adults

**DOI:** 10.1186/s12968-020-00642-y

**Published:** 2020-07-27

**Authors:** Murat Kocaoglu, Amol S. Pednekar, Hui Wang, Tarek Alsaied, Michael D. Taylor, Mantosh S. Rattan

**Affiliations:** 1 Department of Radiology, Cincinnati Children’s Hospital Medical Center, 3333 Burnet Ave, Cincinnati, OH 45229 USA; 2 Department of Radiology, University of Cincinnati College of Medicine, Cincinnati, OH USA; 3 MR Clinical Science, Philips Healthcare, Cincinnati, OH USA; 4 The Heart Institute, Cincinnati Children’s Hospital Medical Center, Cincinnati, OH USA; 5 Department of Pediatrics, University of Cincinnati College of Medicine, Cincinnati, OH USA

**Keywords:** Compressed SENSE, Left ventricular indices, Right ventricular indices, Free-breathing cine, Pediatric, Children

## Abstract

**Background:**

Although the breath-hold cine balanced steady state free precession (bSSFP) imaging is well established for assessment of biventricular volumes and function, shorter breath-hold times or no breath-holds are beneficial in children and severely ill or sedated patients.

**Methods:**

Clinical cardiovascular magnetic resonance (CMR) examinations from September 2019 to October 2019 that included breath-hold (BH) and free-breathing (FB) cine bSSFP imaging accelerated using compressed sensitivity encoding (C-SENSE) factor of 3 in addition to the clinical standard BH cine bSSFP imaging using SENSE factor of 2 were analyzed retrospectively. Patients with structurally normal hearts who could perform consistent BHs were included. Aortic flow measured by phase contrast acquisition was used as a reference for the left ventricular (LV) stroke volume. Comparative analysis was performed for evaluation of biventricular volumes and function, imaging times, quantitative image quality, and qualitative image scoring.

**Results:**

There were 26 patients who underwent all three cine scans during the study period (16.7 ± 6.4 years, body surface area (BSA) 1.6 ± 0.4 m^2^, heart rate 83 ± 7 beats/min). BH durations of 8 ± 1 s with C-SENSE = 3 were significantly shorter (*p* < 0.001) by 33% compared to 12 ± 1 s with SENSE = 2. Actual scan time for BH SENSE (4.9 ± 1.2 min) was comparable to that with FB C-SENSE (5.2 ± 1.5 min; *p*= NS). Biventricular stroke volume and ejection fraction, and LV mass computed using all three sequences were comparable. There was a small but statistically significant (*p* < 0.05) difference in LV end-diastolic volume (− 3.0 ± 6.8 ml) between BH SENSE and FB C-SENSE. There was a small but statistically significant (*p* < 0.005) difference in end-diastolic LV (− 5.0 ± 7.7 ml) and RV (− 6.0 ± 8.5 ml) volume and end-systolic LV (− 3.2 ± 4.3 ml) and RV(− 4.2 ± 6.8 ml) volumes between BH C-SENSE and FB C-SENSE. The LV stroke volumes from all three sequences had excellent correlations (*r* = 0.96, slope = 0.98–1.02) with aortic flow, with overestimation by 2.7 (5%) to 4.6 (8%) ml/beat. The image quality score was Excellent (16 of 26) to Good (10 of 26) with BH SENSE, Excellent (13 of 26) to Good (13 of 26) with BH C-SENSE, and Excellent (3 of 26) to Good (21 of 26) to Adequate (2 of 26) with FB C-SENSE.

**Conclusions:**

Image quality and ventricular volumetric and functional indices using either BH or FB C-SENSE cine bSSFP imaging were comparable to standard BH SENSE cine bSSFP imaging while maintaining nominally identical spatio-temporal resolution. This accelerated image acquisition provides an alternative to accommodate patients with impaired BH capacity.

## Introduction

The assessment of cardiac volumetric indices is important for the diagnosis and follow-up of both congenital and acquired heart disease [1–6]. Cardiovascular magnetic resonance (CMR) imaging is an accurate and reproducible modality that is the clinical reference standard for quantitative evaluation of ventricular chamber size, function and myocardial mass [6–11]. Currently, retrospectively cardiac gated two-dimensional segmented k-space cine balanced steady state free precession (bSSFP) is the preferred CMR sequence for the quantitative assessment of cardiac function. The bSSFP sequence has high intrinsic blood pool to myocardium contrast and high signal-to-noise ratio (SNR) that results in well-defined endocardial boundaries throughout the cardiac cycle [12–14]. Short-axis (Sax) cine bSSFP images are routinely acquired during breath-holds, because the bSSFP sequence is susceptible to artifacts from respiratory motion and disruption of magnetization steady state [14]. In routine clinical practice, one to three cine SAx slices are acquired in a breath-hold (BH) of 5 to15 cardiac cycles by trading the intrinsic high bSSFP signal-to-noise (SNR) for imaging speed using parallel imaging techniques that employ regular k-space undersampling in the spatial dimensions e.g. sensitivity encoding (SENSE), without compromising the blood to myocardial contrast and providing adequate spatio-temporal resolution [15, 16]. Although the accuracy and reproducibility of CMR bSSFP for the measurement of ventricular volumes, function, and cardiac mass is well established [17–19], the requirement for repeated BHs remains a limitation, especially in children and sedated patients. Accelerated cine CMR techniques (such as k-t BLAST, TPAT, TSENSE, and compressed sensing) have reported bias in left ventricular (LV) volumes, function, and LV mass due to spatiotemporal blurring and temporal filtering [20–26]. Free-breathing (FB) respiratory triggered retrospectively cardiac gated cine bSSFP sequences have been reported to provide biventricular volumes, function, and LV mass comparable to BH acquisitions with a SENSE acceleration factor of 2 in adults and children [27, 28]. With the goal of accelerating SENSE, a compressed sensitivity encoding (C-SENSE) algorithm was developed that employs a pseudorandom undersampling of k-space in the spatial domain. C-SENSE provides diagnostic CMR image quality with acceleration factors greater than 2, allowing for significantly reduced BH times [29]. However, there is little data validating quantitative ventricular assessment using C-SENSE either with BH or FB acquisitions [30].

The purpose of our study was to test the hypothesis that the use of C-SENSE acceleration in BH and FB respiratory-gated retrospectively cardiac gated cine bSSFP sequences produces diagnostic quality images and accurate ventricular volumetric indices with decreased BH times.

## Materials and methods

This HIPAA-compliant, retrospective study was approved by the institutional review board (IRB) at our institution. The requirement for informed consent was waived. The free-breathing cine bSSFP sequence in its current form was implemented within our institution, and all the data and information were always under the control of our institution.

### Patients

We identified all patients who had undergone clinically indicated CMR examinations that included BH SENSE, BH C-SENSE, and FB C-SENSE sequences between September 2019 and October 2019. During this period, C-SENSE was used as part of a quality improvement effort to shorten and/or eliminate BH in CMR acquisition protocols. IRB approval was obtained for the current study which involved systematic retrospective review of those images previously obtained for clinical quality improvement. Patients with congenital heart disease and those who could not complete all three scans were not included.

### CMR technique

All CMR examinations were performed with a 1.5 T CMR scanner (Ingenia, Philips Healthcare, Best, The Netherlands). SAx cine bSSFP acquisitions covering the entire heart were performed using vector electrocardiogram gating with a dedicated 28 element torso coil and a respiratory bellows placed at the mediastinum, as in routine clinical CMR sessions. All cine imaging was performed prior to administration of contrast agents. BH cine SAx acquisitions were performed with a SENSE acceleration factor of 2, followed by a second acquisition with a C-SENSE acceleration factor of 3. A third SAx cine acquisition was performed with the cardiorespiratory synchronized [27, 28] FB sequence using Fixed mode (one cardiac cycle per respiration) with a C-SENSE acceleration factor of 3. No special breathing instructions were given during the FB acquisition. All three retrospectively cardiac gated SAx cine acquisitions were performed with identical imaging parameters. The imaging parameters were: repetition time (TR) ms/echo time (TE) ms, 2.5–2.7/1.25–1.35; flip angle (FA), 60°; acquired voxel size, 1.6–1.7 × 1.6–1.7 × 6–8 mm^3^ (zero gap); acquired temporal resolution, 40–45 ms. Actual breath-hold durations and acquisition times were extracted from the scanner log files. As part of the standard clinical protocol, quantitative flow assessment (TR/TE - 4.5/2.7; FA, 12°; acquired voxel size, 1.6–1.7 × 1.6–1.7 × 6 mm^3^; acquired temporal resolution, 40–45 ms; velocity encoding, 150–200 cm/s) of the aorta at the level of sinotubular junction was performed.

Commercially available implementation of SENSE and C-SENSE reconstruction were used. The SENSE algorithm employs data consistency based on a regular undersampling pattern and coil sensitivity information, and spatial solution space constraint based on prior knowledge of the image extent [31]. The C-SENSE combines a spatial domain pseudo-random undersampling pattern of k-space with the SENSE reconstruction algorithm using iterative reconstruction and sparsity constraints [31]. Both these techniques required coil sensitivity and noise estimation information from data acquired during the pre-scan. The 3D pre-scan with coil specific field of view and spatial resolution is performed in a single 7 s breath-hold, equivalent to single slice acquisition, and information is used for the entire SAx stack. No additional BH is incurred if pre-scan information collected for the localizers and previous scans is compatible with the field of view prescribed for the cine SAx stack and table and patient position is unchanged. The regularization parameters are automatically adjusted for individual patient body habitus, coil topology, and SNR of the prescribed sequence. Specifically, for cine bSSFP sequence, the transient phase for bSSFP is initialized by an α/2 – TR/2 preparation followed by an alternating radiofrequency phase scheme that generates a steady-state [14, 32]. The ky undersampling pattern is determined by a pseudo-random variable density Poisson distribution for the prescribed field of view and spatial resolution. Based on the prescribed temporal resolution of a single cardiac phase, this ky pattern is then divided into multiple sequential k-space segments of an equal number of ky lines. Thus, the phase encoding gradient amplitude change is minimal close to the center of k-space and signal instabilities due to eddy currents are confined to the periphery of k-space [32]. The k-space segment is repeated for each cardiac phase within a cardiac cycle and subsequently used for retrospective cardiac gating.

### Image analysis

All images were transferred to a separate post-processing workstation (Medis Suite 3.1, Medis Medical Imaging Systems, Leiden, The Netherlands). The SAx cine images were analyzed by a single reviewer (MK), under the supervision of a cardiac radiologist (MR) with > 6 years of experience who reviewed all measurements. The quantitative assessment of the LV and right ventricular (RV) volumetric indices (end-diastolic volume, end-systolic volume, stroke volume, ejection fraction) and LV mass was performed using the SAx cine bSSFP images. End diastolic and end systolic phases were defined at the midventricular level. For all SAx cine bSSFP series LV basal slices were defined when at least 50% of the myocardium was visible next to the mitral valve [33, 34]. The RV basal slice was defined below the pulmonary valve and the inflow tract areas were excluded if surrounding myocardial muscle was thin and not trabeculated, suggestive of right atrium [35]. The apical ventricular slices were defined as the last slice showing intracavity blood pool. All endocardial and epicardial contours were drawn manually. The papillary muscles were not contoured and were assigned to the ventricular cavities [36]. Regions of interests were drawn inside the LV blood pool and septal myocardium in diastole to compute the normalized blood-to-myocardial contrast. Image quality was graded independently by three CMR readers (MK, MR and TA with > 3 years’ experience in CMR). The image quality scores were based on three criteria: blood-to-myocardial contrast (BMC), endocardial edge delineation (EED), and presence of artifacts such as bulk motion artifacts and residual undersampling related artifacts. Each criterion was graded on a scale of 1 to 5, where 1 was nondiagnostic, 2 was suboptimal but still diagnostic for volumetric analysis, 3 was adequate, 4 was good, and 5 was excellent. All the slices were reviewed individually and an average image quality score for each criterion was assigned to the entire SAx stack. The total combined image quality score was calculated as the average of the three scores. All the patient data were included in the analysis.

### Data analysis

Descriptive statistics of continuous quantitative measurements were summarized as means and standard deviations. Bland-Altman analysis [37] and the two-sided paired *t* test were used to compare each of the parameters computed using standard of care BH SENSE with those computed using BH and FB C-SENSE acquisitions. LV stroke volume computed using SAx cine imaging and aortic quantitative flow were compared using Bland-Altman analysis. A *p*-value < 0.05 was considered significant for all inference testing and 95% confidence intervals were calculated as appropriate. Tukey multiple comparison analyses and Tukey box plots [38] were used to compare scan time, BMC normalized to myocardial signal, and image quality scores between three cine bSSFP acquisitions. The Kruskal-Wallis test was performed to compare differences in image quality scores between readers and between scoring criteria. Wilcoxon signed-rank tests were performed to compare image quality scores assigned to the BH SENSE, BH C-SENSE, and FB C-SENSE acquisitions. For each of the three image quality scoring criteria considered in the study, the percentage of clinical subjects who received a range of image quality scores was plotted as a bar graph. All statistical analyses were performed using MATLAB (The MathWorks™ Inc., Natick, Massachusetts, USA).

## Results

**Fig. 1:**
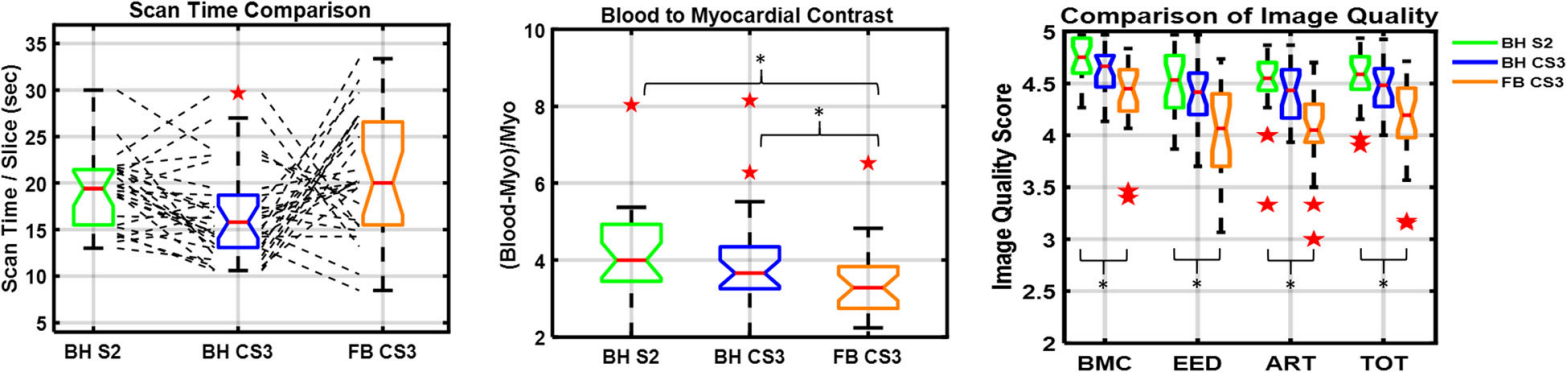
Tukey box plots of (**a**) imaging duration, (**b**) blood-to-myocardial contrast (BMC) normalized to myocardial signal, and (**c**) image quality scores for three short-axis acquisitions. Imaging duration includes gaps between breath-holds. One-to-one line plots (dotted black lines) for imaging duration with breath hold (BH) C-SENSE = 3 and free breathing (FB) C-SENSE = 3 depict the dependence of imaging duration on the patient’s heart rate–to–respiratory rate ratio. BMC of FB C-SENSE = 3 scan was significantly lower (*p* < 0.05) than both BH SENSE = 2 and BH C-SENSE = 3 acquisitions. Center red line = median, whiskers = minimum and maximum within 1.5 times interquartile distance, red ★ = outliers beyond 1.5 times the interquartile distance. Non-overlapping notches indicate that the medians of the two groups differ at the 5% significance level. Black * with bracket indicates two groups are significantly different (*p* < 0.05). ART = artifacts; CS3 C-SENSE acceleration factor of 3; EED = endocardial edge delineation; S2 SENSE acceleration factor of 2; TOT = total combined score

There were 26 patients (26 males; 16.7 ± 6.4 years (range: 9–35), body surface area (BSA) 1.6 ± 0.4 (range: 0.94–2.4 m^2^) and heart rate 83 ± 7.2 beats/min (range: 50–115)) who underwent all three SAx cine scans during the study period. Table 1 summarizes the patient characteristics. Indications for CMR included Duchenne muscular dystrophy (*n* = 12), pectus excavatum (*n* = 10), Becker muscular dystrophy (*n* = 1), Marfan syndrome (*n* = 1), Turner syndrome (*n* = 1), and chemotherapy induced cardiomyopathy (*n* = 1). All SAx cine images were obtained without technical failure or significant artifact. A total of 15 ± 2 (range: 12–18) SAx slices were acquired per patient in 8 ± 1 breath-holds (2 slices per breath-hold) (range: 6–9). BH durations of 8 ± 1 (range: 7–10) sec with C-SENSE = 3 were significantly shorter (*p* < 0.001) by 33% compared to 12 ± 1 s (range: 10–14) with SENSE = 2. Actual image acquisition time, including BH instructions and time between BHs, for the SAx stack with BH SENSE = 2 (4.9 ± 1.2 min (range: 2.6–7)) was significantly longer (*p* < 0.05) compared to that with BH C-SENSE = 3 (4.3 ± 1.5 min (range: 2.1–7)) and comparable to that with FB C-SENSE = 3 (5.2 ± 1.5 min (range: 2.3–7)). Figure 1a depicts the comparison of scan time per slice.

**Table 1:** Characteristics of the study population

Number of patients	26
Age (years)	16.7 ± 6.4 (9–35)
Female-to-male ratio	6:20
Height (cm)	154.3 ± 21.9 (121–191)
Weight (kg)	61 ± 26 (24–134)
BSA (m^2^)	1.59 ± 0.38 (0.94–2.44)
Heart rate (beats/min)	83 ± 17 (50–115)
Clinical Indications	
Duchenne muscular dystrophy	12
Pectus excavatum	10
Becker muscular dystrophy	1
Marfan syndrome	1
Turner syndrome	1
Chemotherapy induced cardiomyopathy	1

The data are presented as the mean ± standard deviation (minimum – maximum) or as the number of subjects

*BSA* body surface area

**Fig. 2:**
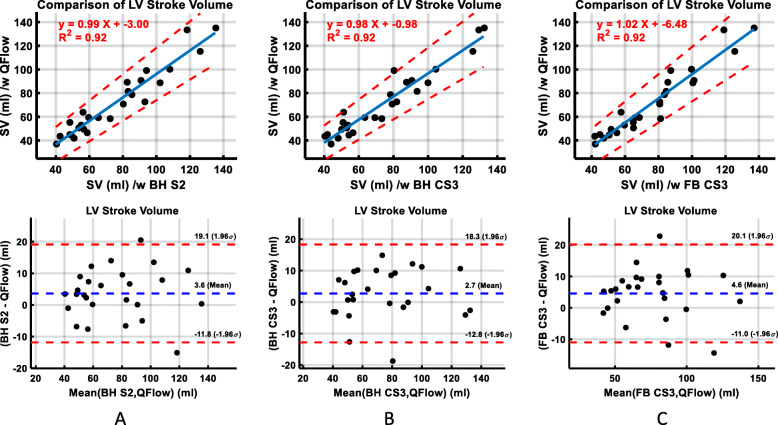
Linear regression and Bland-Altman plots comparing left ventricular (LV) stroke volume (SV) measured with aortic quantitative flow with that measured from bSSFP cine short-axis images acquired with (**a**) BH SENSE = 2, (**b**) BH C-SENSE = 3, and BH breath-hold, CS3 C-SENSE acceleration factor of 3, FB free-breathing, S2 SENSE acceleration factor of 2

Table 2 demonstrates the data comparing LV and RV volumetric indices measured with the three sequences. LV and RV stroke volume and ejection fraction, and LV mass computed were comparable. There were no significant differences in RV volumes, and LV and RV stroke volumes, ejection fractions, or myocardial mass between BH SENSE and BH C-SENSE sequences. There was a small but statistically significant difference in LV and RV ventricular end diastolic and end systolic volumes between BH C-SENSE and FB C-SENSE sequences. Figure 2 depicts linear regression and Bland-Altman plots comparing LV stroke volume computed using aortic quantitative flow with those computed using three cine bSSFP SAx acquisitions. The LV stroke volumes from the three sequences had excellent correlations with aortic flow, regression slopes ranged from 0.98 to 1.02. All three sequences overestimated the LV stroke volume by 2.7 (5%) to 4.6 (8%) ml/beat compared with the aortic phase contrast data. The limits of agreement of LV stroke volumes for all three sequences was less than 24%.

**Table 2:** Left and Right Ventricular Volumetric Indices and Difference in their Values between Breath-hold with SENSE Acceleration Factor of 2, Breath-hold with C-SENSE Acceleration Factor of 3, and Free-breathing Sequence with C-SENSE Acceleration Factor of 3 (All Subjects)

	Imaging Sequence			Difference			***P*** value		
	BH S2	BH CS3	FB CS3	BH S2 - BH CS3	BH S2 - FB CS3	BH CS3 - FB CS3	BH S2 - BH CS3	BH S2 - FB CS3	BH CS3 - FB CS3
**LV EDV (ml)**	127.2 ± 46.2	125.2 ± 45.8	130.2 ± 45.9	2.0 ± 6.9	−3.0 ± 6.8	−5.0 ± 7.7	0.142	0.036*	0.003*
**LV EDV / BSA**	79.3 ± 18.7	77.8 ± 17.5	81.0 ± 17.6	1.5 ± 4.7	−1.8 ± 4.2	−3.3 ± 5.1	0.122	0.043*	0.003*
**LV ESV (ml)**	52.0 ± 21.7	50.8 ± 21.2	54.0 ± 22.6	1.2 ± 4.4	−2.0 ± 4.8	−3.2 ± 4.3	0.191	0.042*	< 0.001*
**LV ESV / BSA**	32.2 ± 9.4	31.4 ± 8.6	33.4 ± 9.3	0.8 ± 3.0	−1.1 ± 2.9	−2.0 ± 2.7	0.184	0.058	0.001*
**LV SV**	75.3 ± 27.0	74.4 ± 27.4	76.2 ± 26.1	0.9 ± 5.8	−0.9 ± 6.4	−1.8 ± 6.0	0.440	0.463	0.131
**LV SV / BSA**	47.0 ± 11.4	46.4 ± 11.2	47.7 ± 10.8	0.7 ± 3.6	−0.6 ± 4.2	−1.3 ± 3.9	0.351	0.458	0.098
**LV EF (%)**	59.6 ± 5.5	59.7 ± 6.1	59.1 ± 6.2	−0.2 ± 2.8	0.5 ± 3.5	0.7 ± 2.4	0.748	0.480	0.172
**LV Mass (g)**	63.2 ± 20.3	65.2 ± 20.9	63.6 ± 21.0	−2.0 ± 4.3	−0.4 ± 7.3	1.6 ± 6.6	0.026*	0.764	0.237
**LV Mass / BSA**	39.4 ± 7.2	40.5 ± 7.0	39.6 ± 7.8	−1.1 ± 2.9	−0.2 ± 4.6	1.0 ± 4.0	0.063	0.854	0.237
**RV EDV (ml)**	121.1 ± 44.6	117.9 ± 43.5	123.9 ± 45.8	3.2 ± 6.2	−2.8 ± 9.6	−6.0 ± 8.5	0.015*	0.153	0.001*
**RV EDV / BSA**	75.6 ± 19.8	73.3 ± 18.1	77.2 ± 19.8	2.3 ± 4.2	−1.6 ± 6.0	−3.9 ± 5.7	0.009*	0.196	0.002*
**RV ESV (ml)**	51.9 ± 22.6	48.7 ± 20.4	52.9 ± 22.5	3.2 ± 5.5	−1.0 ± 5.9	−4.2 ± 6.8	0.007*	0.384	0.004*
**RV ESV / BSA**	32.1 ± 11.2	30.1 ± 9.7	32.8 ± 11.3	2.0 ± 3.7	−0.7 ± 3.9	−2.7 ± 4.8	0.010*	0.391	0.008*
**RV SV**	69.3 ± 24.2	69.2 ± 24.5	71.0 ± 25.0	0.0 ± 5.3	−1.7 ± 5.9	−1.8 ± 3.9	0.985	0.144	0.030*
**RV SV / BSA**	43.5 ± 10.6	43.2 ± 9.5	44.4 ± 10.0	0.3 ± 3.1	−0.9 ± 3.5	−1.2 ± 2.5	0.621	0.203	0.024*
**RV EF (%)**	58.1 ± 5.9	59.5 ± 4.8	58.2 ± 5.9	−1.4 ± 3.5	−0.1 ± 2.9	1.2 ± 3.6	0.054	0.820	0.094

Unless otherwise indicated, data are means ± standard deviations

*BH* breath-hold, *BSA* body surface area, *CS3* C-SENSE acceleration factor of 3, *EDV* end-diastolic volume, *EF* ejection fraction, *ESV* end-systolic volume, *FB* free-breathing, *LV* left ventricle, *RV* right ventricle, *S2* SENSE acceleration factor of 2, *SV* stroke volume

The * represents *p* < 0.05

**Fig. 3:**
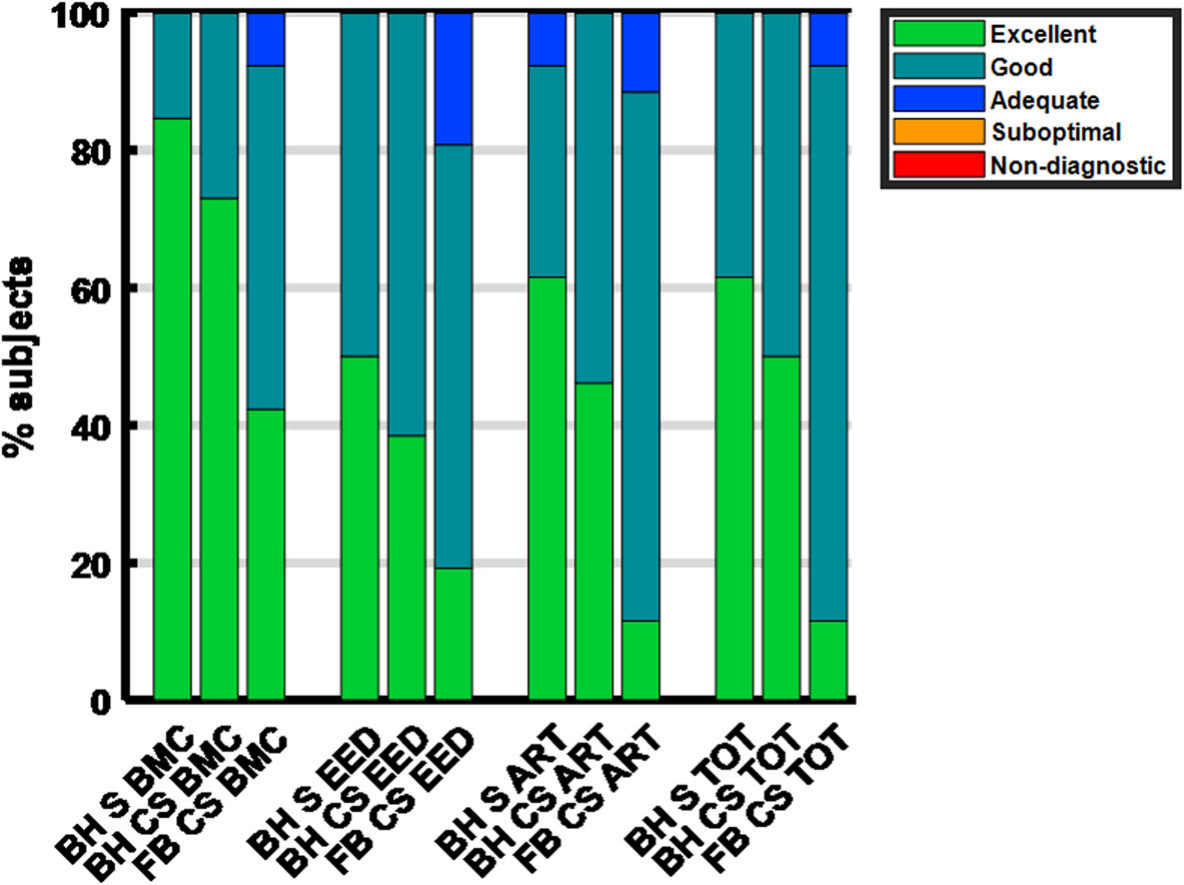
Bar-plot analysis of image quality scores depicts percentage of patients who had image quality scores of excellent, good, adequate, suboptimal, or non-diagnostic in each grading criteria based on blood-to-myocardial contrast (BMC), endocardial edge definition (EED), and presence of artifacts (ART) and total (TOT) combined score. The combined image quality score is the equal-weight average of the three scores, which underscores the overall performance of the technique

**Fig. 4:**
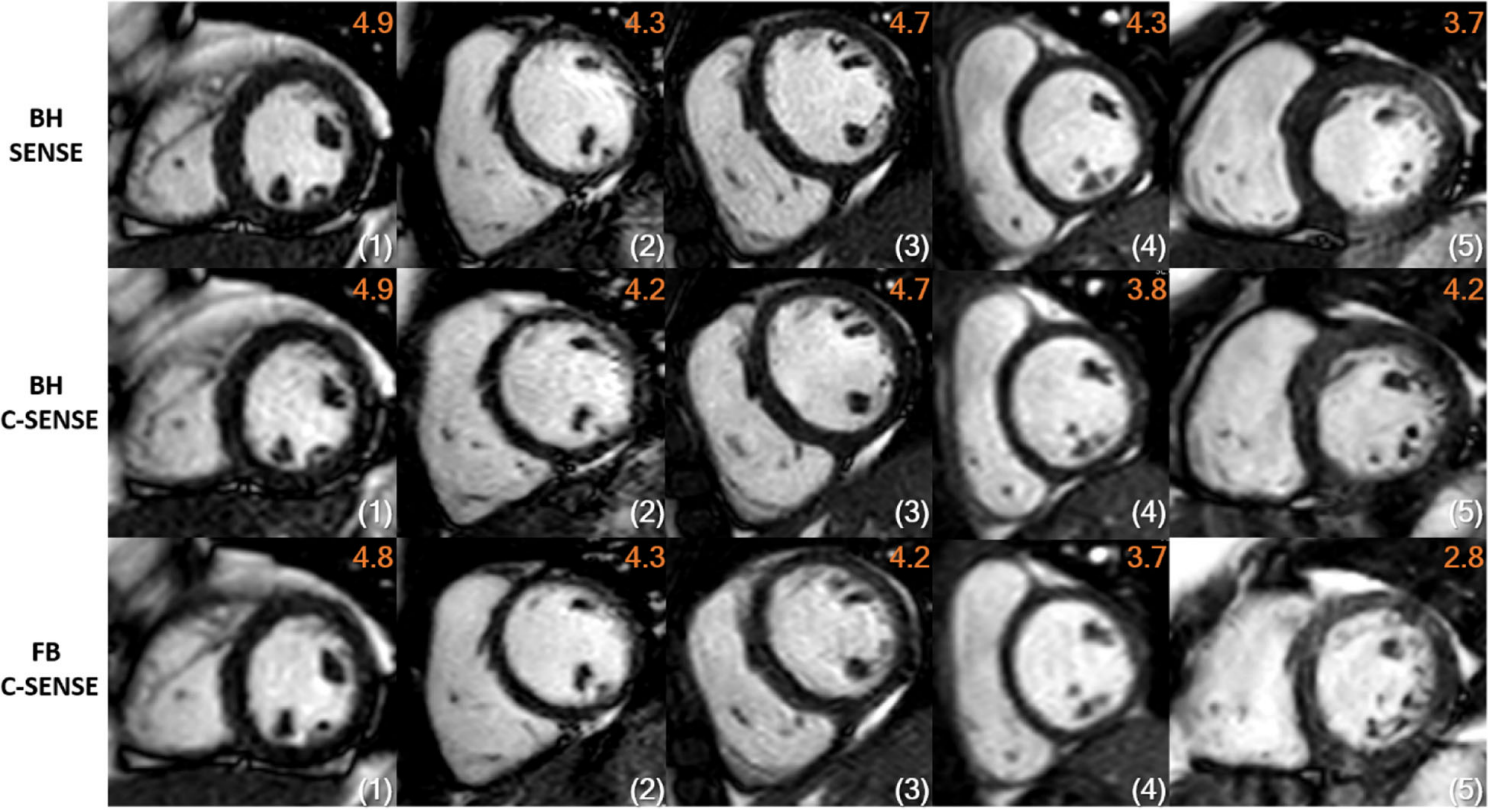
Representative balanced steady-state free precession short-axis images of 5 patients (columns) with combined clinical scores: breath-hold (BH) with SENSE factor of 2 (top row), breath-hold with C-SENSE factor of 3 (middle row), and free-breathing (FB) with C-SENSE factor of 3(bottom row). The combined image quality score is the equal-weight average of score in three criteria: blood-to-myocardial contrast (BMC), endocardial edge definition (EED), and presence of artifacts throughout the cardiac cycle. Each criterion was graded on a scale of 1 to 5, where 1 is nondiagnostic, 2 is suboptimal but still diagnostic for volumetric analysis, 3 is adequate, 4 is good, and 5 is excellent. Patient in (1) had dark papillary muscles and endocardial trabeculae clearly visible with crisp edge definition on bright backdrop of the blood pool throughout the cardiac cycle in all slices in all the three sequences. Patient in (2) had mild degradation of edge definition in all three sequences. For the patient in (3), there was mild degradation of edge definition only in FB C-SENSE sequence in couple of slices. Patient in (4) had mild degradation of edge definition for all three sequences, additionally both BH C-SENSE and FB C-SENSE sequences had slightly less BMC. For patient in (5), there were mild parallel imaging and motion artifacts for BH SENSE sequence, while BH C-SENSE sequence had only mild degradation of the edge definition, and FB C-SENSE sequences had substantial motion artifacts. BH = breath-hold, FB = free-breathing cardiorespiratory synchronized retrospectively cardiac gated balanced steady-state free precession cine CMR sequence

BMC normalized to myocardial signal for FB C-SENSE was significantly lower (*p* < 0.05) than with both BH SENSE and BH C-SENSE acquisitions (Fig. 1b). Image quality scores were comparable between the three readers across all criteria in all sequences. The mean of three observers’ scores in each criterion were used for further analysis. Mean rank scores for BMC, EED, artifacts, and total image quality score with FB C-SENSE acquisitions were significantly lower (*p* < 0.005) than with the BH SENSE acquisitions (Fig. 1c). Mean rank scores for EED and combined total image quality scores with BH C-SENSE were significantly lower (*p* < 0.05) than BH SENSE. Mean rank scores in each scoring criterion were comparable between BH SENSE and BH C-SENSE sequences. Figure 3 depicts the image quality scores for each scoring criteria. The combined image quality score was excellent (16 of 26) to good (10 of 26) with BH SENSE, excellent (13 of 26) to good (13 of 26) with BH C-SENSE, and excellent (3 of 26) to good (21 of 26) to adequate (2 of 26) with FB C-SENSE (Fig. 3). For the combined image quality score, the difference between BH SENSE and BH C-SENSE was (0.08 ± 0.18 (range: − 0.50 - 0.39) and the difference between BH SENSE and FB C-SENSE was (0.42 ± 0.34 (range: − 0.26 - 1.17). Figure 4 shows representative images with excellent, good, and adequate combined image quality scores using all three acquisition techniques.

## Discussion

The results of this retrospective study demonstrated that image quality and biventricular volumetric indices using cine bSSFP acquisition with C-SENSE acceleration factor of 3, either during BH or FB, are comparable to the standard of care BH cine bSSFP acquisition with SENSE acceleration factor of 2. Both BH and FB cine bSSFP sequences with C-SENSE = 3 had spatio-temporal resolution nominally identical to the standard of care BH sequence and provided diagnostic image quality in all 26 patients encompassing a wide range of body sizes and heart rates. The BH duration was reduced by 33% using C-SENSE = 3 compared to SENSE = 2. The total imaging time for the FB acquisition with C-SENSE = 3 was comparable to SENSE = 2.

Although the standard BH cine bSSFP sequences are well established, accelerated acquisition with shorter BH times or without BHs helps with imaging children and severely ill or sedated patients. Numerous strategies for undersampling k-space in the time domain using either regular or irregular patterns in combination with either prospective cardiac gating or real-time cine imaging have been reported to provide diagnostic image quality and comparable ventricular volumetric assessment [20, 21, 23–26, 39–43]. However, studies with temporal undersampling schemes have reported underestimation of the LV mass and bias in both stroke volume and ejection fraction [23–26]. Some of the difference in volumetric indices can be attributed to experimental and physiologic variations. However, bias in volumetric measurements is partly due to the decrease in effective temporal resolution when using temporal undersampling. Systematic evaluation of incremental increases in temporal undersampling using different reconstruction approaches on fully sampled cine bSSFP data has shown that these biases worsen with increasing acceleration factors and can be larger than physiologic variations [44]. In our study, bias and standard deviations for volumetric indices of both BH and FB C-SENSE = 3 sequences compared to standard of care BH SENSE = 2 sequence are comparable to the inter- and intra-observer values reported in the literature [19, 26, 35, 45–47]. The overestimation of LV stroke volume by SAx measurements compared to aortic flow is 4 to 7% of the LV mass, which is comparable to reported LV trabeculation mass value of 8.2% seen in healthy subjects [48]. A consistent, small, non-zero velocity offset in aortic phase contrast data due to double-oblique slice orientation at the sinotubular junction may also have contributed to this difference. Overall, the statistically significant but small absolute difference seen in ventricular volumes between BH and FB is not clinically significant.

Although biventricular volumetric indices and LV mass were comparable across the three acquisitions, the endocardial delineation of both the BH and FB C-SENSE acquisitions were slightly inferior to BH SENSE. This suggests that spatial blurring caused by spatial domain pseudorandom sampling likely contributes to the differences in RV volumetric indices, exacerbated by the irregular RV contour shape. Decrease in blood-to-myocardial contrast can be attributed to diminished blood pool signal due to increased undersampling. The differences in BMC image quality between BH and FB C-SENSE suggest inferior attainment of steady state in FB sequence due to longitudinal through plane motion during the cardiac cycle, particularly in ventricular basal slices. The variability in depth of breathing may also contribute to the decrease in EED and artifact scores for FB compared to BH C-SENSE. This is noted frequently in our patients with muscular dystrophy and pectus excavatum. Another artifact noticed in larger patients was residual parallel lines next to bSSFP black bands at the edges of the field of view in images with C-SENSE factor of 3.

In addition to the noise penalty associated with the undersampling, the noise is further amplified in parallel imaging due to the nonorthogonality of the coil sensitivity profiles reflected by geometry or g-factors [49]. In 2D cartesian parallel imaging techniques, the image degradation due to noise increases exponentially around critical reduction factor of 3 to 4 [50]. A recent study in healthy adults using C-SENSE factor of 4 for cine bSSFP imaging showed adequate image quality; however, the acquired voxel size of 2.8 mm in phase encode direction was significantly larger than 1.6–1.7 mm used in our pediatric population [30]. Additionally, that study depended on shallow breathing while our study used explicit respiratory gating. In order to minimize the spatial blurring that occurs with iterative reconstruction from pseudo randomly sampled k-space data while trying to accelerate image acquisition, we conservatively tested a C-SENSE factor of 3 in this study. This study showed that the C-SENSE factor of 3 shortened the breath-hold time for SAx cine bSSFP acquisitions significantly while maintaining adequate LV and RV myocardial border definition. We showed good agreement of volumetric indices with those acquired with SENSE factor of 2. This reduction in acquisition time can be traded for either faster patient throughput by acquiring additional slices per BH or to accommodate patients with impaired BH capacity.

Furthermore, the study demonstrated that C-SENSE acquisitions can be used for quantitative ventricular assessment along with FB cardiorespiratory synchronized cine bSSFP. Although study population consisted of children and young adults with structurally normal hearts, the sequence has potential utility for FB cardiorespiratory synchronized cine bSSFP in combination with C-SENSE in older adults as well, especially patients with impaired BH capacity or capability. There is nothing about the sequence or acquisition scheme that would preclude large or older patients.

There are few limitations to this study. First, we did not acquire the cine bSSFP data with full k-space sampling. Quantitative aortic flow was used as an internal reference to partially address this limitation. Second, the study population with structurally normal hearts mostly consisted of pectus and muscular dystrophy patients, both of which have male predominance. Third, the data acquisition for this retrospective study was performed as part of clinical scan sessions, so actual scan time comparisons between BH acquisitions is confounded by the irregular gaps between consecutive BHs. Lastly, SNR between different acquisitions was not compared quantitatively given the complex spatial distribution of noise in C-SENSE due to both data pseudorandom acquisition and iterative reconstruction. Differences in SNR were partially incorporated in the BMC measurements.

## Conclusion

Cine bSSFP imaging with compressed SENSE acceleration factor of 3 reduces BH times by 33% compared to a clinically established SENSE acceleration factor of 2 with nominally identical spatio-temporal resolution while providing comparable LV and RV volumetric and functional indices and image quality. Also, C-SENSE combined with cardiorespiratory synchronized free-breathing cine bSSFP imaging provides comparable LV and RV volumetric and functional indices to that with clinically established BH acquisition with SENSE factor of 2 in comparable time. This reduction in acquisition time can be traded for either faster scan sessions with more slices per breath-hold or for reduction or elimination of BHs to accommodate patients with impaired BH capacity.

## Supplementary information

**Additional file 1.**

**Additional file 2.**

**Additional file 3.**

**Additional file 4.**

**Additional file 5.**

**Additional file 6.**

## Data Availability

The datasets generated and/or analyzed during the current study are not publicly available due to patient privacy concern and institutional policies but are available from the corresponding author on reasonable request.

## Abbreviations

BH: Breath hold
BMC: Blood-to-myocardial contrast
BSA: Body surface area
bSSFP: Balanced steady state free precession
C-SENSE: Compressed sensitivity encoding
CMR: Cardiovascular magnetic resonance
EDV: End-diastlic volume
EED: Endocardial edge definition
FA: Flip angle
FB: Free breathing
LV: Left ventricle/left ventricular
RV: Right ventricle/right ventricular
SAx: Short axis
SENSE: Sensitivity encoding
SNR: Signal-to-noise ratio
TE: Echo time
TR: Repetition time
