# Supplementary figures and images for: Breath-hold and free-breathing quantitative assessment of biventricular volume and function using compressed SENSE: a clinical validation in children and young adults
Source: J Cardiovasc Magn Reson. 2020 Jul 27;22:54. doi: 10.1186/s12968-020-00642-y (PMC7384228; doi:10.1186/s12968-020-00642-y)

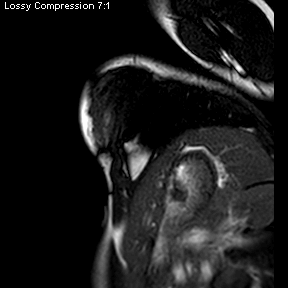

Supplement: Supplementary file 1 — Additional file 1. [file 12968_2020_642_MOESM1_ESM.gif]

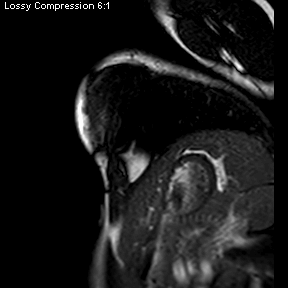

Supplement: Supplementary file 2 — Additional file 2. [file 12968_2020_642_MOESM2_ESM.gif]

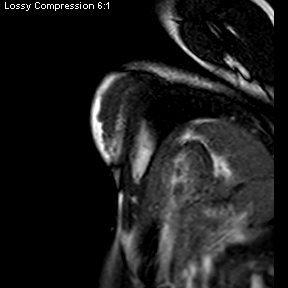

Supplement: Supplementary file 3 — Additional file 3. [file 12968_2020_642_MOESM3_ESM.gif]

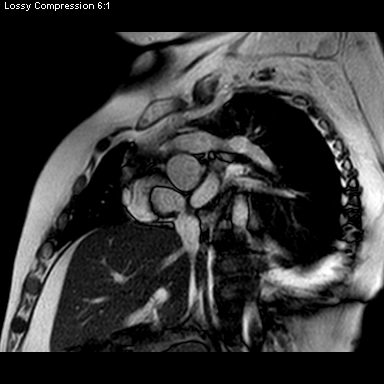

Supplement: Supplementary file 4 — Additional file 4. [file 12968_2020_642_MOESM4_ESM.gif]

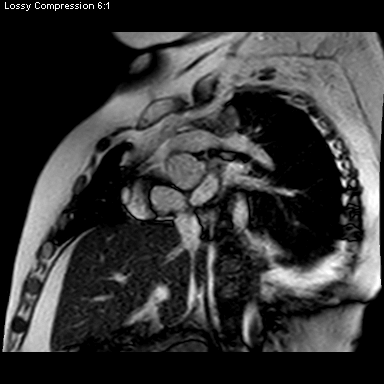

Supplement: Supplementary file 5 — Additional file 5. [file 12968_2020_642_MOESM5_ESM.gif]

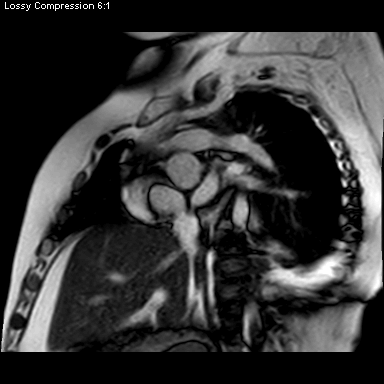

Supplement: Supplementary file 6 — Additional file 6. [file 12968_2020_642_MOESM6_ESM.gif]
